# Pancharatnam–Berry phase reversal via opposite-chirality-coexisted superstructures

**DOI:** 10.1038/s41377-022-00835-3

**Published:** 2022-05-12

**Authors:** Lin Zhu, Chun-Ting Xu, Peng Chen, Yi-Heng Zhang, Si-Jia Liu, Quan-Ming Chen, Shi-Jun Ge, Wei Hu, Yan-Qing Lu

**Affiliations:** National Laboratory of Solid State Microstructures, Key Laboratory of Intelligent Optical Sensing and Manipulation, College of Engineering and Applied Sciences, and Collaborative Innovation Center of Advanced Microstructures, Nanjing University, 210093 Nanjing, China

**Keywords:** Liquid crystals, Photonic crystals, Photonic devices

## Abstract

Recently discovered reflective Pancharatnam–Berry phase (PB phase) from chiral anisotropic media (e.g., cholesteric liquid crystal, CLC) has aroused great interest in the emerging frontier of planar optics. However, the single chirality of common CLCs results in the intrinsic limitation of the same spin-selective PB phase manipulation, which means the reversal of the input spin cannot realize the conjugated PB phase. In this work, an innovative scheme based on opposite-chirality-coexisted superstructures is proposed to simultaneously modulate orthogonal circular polarization and get PB phase reversal. Through refilling CLC into a washed-out polymer network with opposite chirality and delicate photo-patterned structures, reflective optical vortex (OV) with opposite topological charges and vector beams with conjugated spiral PB phases are efficiently generated depending on the incident polarization. Furthermore, OV holograms are encoded to reconstruct polarization-selective OV arrays, indicating the strong capability of such opposite-chirality-coexisted anisotropic media. This work provides a new compact platform for planar optics, and sheds light on the architectures and functionalities of chiral superstructures.

## Abstract (web summary)

The intrinsic limitation of single spin-selective Pancharatnam-Berry phase in common chiral anisotropic media has been broken by a refilled polymer network with opposite chirality and delicate photo-patterned superstructures.

## Graphical abstract

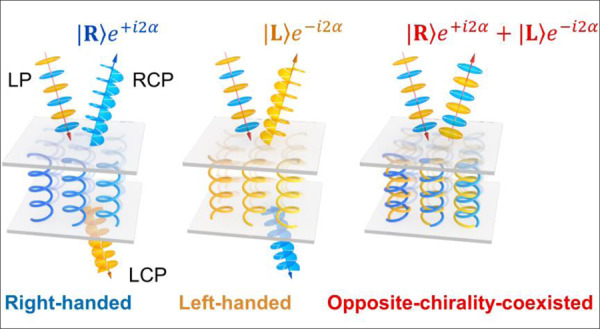

## Introduction

On-demand multi-dimensional optical field tailoring, including the modulation of amplitude, phase, spin angular momentum (SAM) related to polarization, and orbital angular momentum (OAM) related to spiral phase, is essential in photonic applications. In recent years, planar optical elements have garnered great attention due to their flat and compact characteristics^1,2^. As an important branch of the fundamental physics of flat optics, Pancharatnam–Berry phase (PB phase)^3^, also known as the geometric phase, originates from the symmetry breaking in spin–momentum space and the spin–orbit interaction^4,5^. It is usually generated via inhomogeneous anisotropic materials, especially metasurfaces^6–8^ featured by the subwavelength scale and ultra-compatibility. However, challenges still remain in the realization of cost-efficient large-scale fabrication, high optical efficiency, and reliable dynamic light control. In this aspect, liquid crystal (LC) becomes a strong candidate thanks to its high sensibility to external stimuli and various light-matter interaction^9,10^. Nematic LC has been extensively investigated, inspiring versatile PB phase devices in augmented/virtual reality displays^11,12^ and beam steering^13,14^, but half-wave condition should be satisfied to optimize the optical effect.

Cholesteric liquid crystal (CLC), another typical LC mesophase, is attractive as the self-assembled soft photonic crystal with chiral superstructures^15,16^. An intriguing spin-determined Bragg reflection (i.e., photonic band gap) is exhibited^17^, within which the incident light with the same chirality as the CLC helical structures will be reflected while the opposite one is transmitted. Till 2016, it was found that such reflective light carries an additional PB phase associated with CLC helix orientations^18–20^. Light control with broadband equivalent efficiency can be realized regardless of any half-wave condition^21–23^. Particularly, the sensitivity of CLC materials to multiple stimuli (e.g., heat, electric/magnetic field, and light irradiation)^24–27^ makes multi-functional and active PB phase elements possible^28–30^. However, the single-handed chiral structure of CLC determines that the reversal of the input light polarization does not mean the same as the PB phase^31^. In another word, common CLC cannot manipulate light with opposite SAM simultaneously, let alone get the conjugated PB phase. In order to break this limitation, stacking CLC layers with opposite handedness was demonstrated^32^. More recently, taking advantage of the SAM reversal by the mirror and SAM preservation by CLC, a smart approach has been proposed by adding a mirror at the back of CLC device^31^. These works enlighten scientists to seek out a more compact scheme exempted from careful alignment. Then, it comes to us whether it is possible to break the intrinsic single chirality of common CLCs, thus realizing simultaneous PB phase control for both SAMs. Luckily, the CLC polymer networks reported in prior arts^33–36^, which are compatible with media with different properties, guide our way.

In this work, we propose an innovative scheme for the simultaneous manipulation of the orthogonal spin-selective PB phase via opposite-chirality-coexisted anisotropic media. This special system is demonstrated by refilling CLC into a washed-out polymer network with opposite chirality. Corresponding transmittance spectra verify that the final opposite-chirality-coexisted structure reflects both circularly polarized light. As a typical example, the azimuthally variant pattern is imprinted into such CLC chiral superstructures through photo-alignment technology. Accordingly, for orthogonal circularly polarized incident light, reflective optical vortex (OV)^37^ with opposite topological charge is generated. While for different linearly polarized light, vector beams with various polarization distributions are created in the reflection. To further verify the capability of the proposed compact formalism for arbitrary PB phase modulation, an OV hologram to create polarization-dependent OV arrays is exhibited with satisfactory performances. This work breaks the limitation of traditional CLC PB phase devices and brings important insight into the chiral-anisotropic-media-mediated polarization optics and photonics.

## Results

### Design principle

**Fig. 1:**
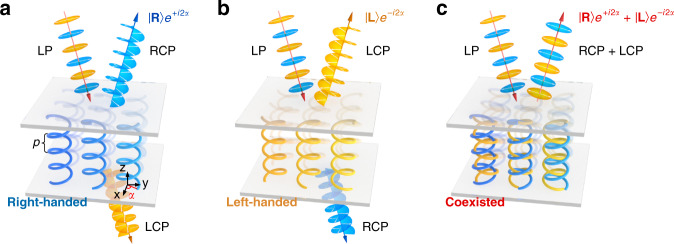
Schematic illustration of common CLC superstructures and opposite-chirality-coexisted superstructures. **a** With the incident light of linear polarization (LP), for right-handed CLC, the right circular polarization (RCP) component is reflected and added by a reflective PB phase of +2*α* (i.e., $\left| {{{\mathbf{R}}}} \right\rangle {{{\mathrm{e}}}}^{ + i2\alpha }$), while left circular polarization (LCP) component is transmitted*. α* represents the orientation angle of the surface LC director. *p* stands for the helical pitch of the CLC. **b** For left-handed CLC, the LCP component is reflected and added by a reflective PB phase of *-*2*α* (i.e., $\left| {{{\mathbf{L}}}} \right\rangle {{{\mathrm{e}}}}^{ - i2\alpha }$), while the RCP component is transmitted. **c** For opposite-chirality-coexisted superstructures, both the RCP and the LCP components are reflected and added by the conjugated PB phase (i.e., $\left| {{{\mathbf{R}}}} \right\rangle {{{\mathrm{e}}}}^{ + i2\alpha } + \left| {{{\mathbf{L}}}} \right\rangle {{{\mathrm{e}}}}^{ - i2\alpha }$)

Spin-selective broadband Bragg reflection is the most important characteristic of CLC chiral superstructure. When the light with the same polarization helicity as the CLC propagates along the CLC helical axis, it will be reflected over a wavelength range of *n*_o_*p-n*_e_*p*^17^, where *p* is the helical pitch and *n*_o_/*n*_e_ are the ordinary/extraordinary refractive indices, respectively. Through designing the orientation distribution of surface LC director (*α*), PB phase of ±2*α* can be encoded into the reflected light, depending on the helicity of both incident light and CLC superstructures^18–20^. As depicted in Fig. 1a, for the linear polarization (LP), right-handed CLC reflects the right circular polarization (RCP) component and a reflective PB phase of +2*α* is imprinted ($\left| {{{\mathbf{R}}}} \right\rangle {{{\mathrm{e}}}}^{ + i2\alpha }$), while left circular polarization (LCP) is transmitted without PB phase modulation^18–20^. On the contrary, left-handed CLC reflects the LCP component added by −2*α* PB phase ($\left| {{{\mathbf{L}}}} \right\rangle {{{\mathrm{e}}}}^{ - i2\alpha }$), and the RCP is transmitted (Fig. 1b). Therefore, for common CLCs, only one circular polarization can be reflected and the reversal of the input SAM does not get the same of the PB phase.

**Fig. 2:**
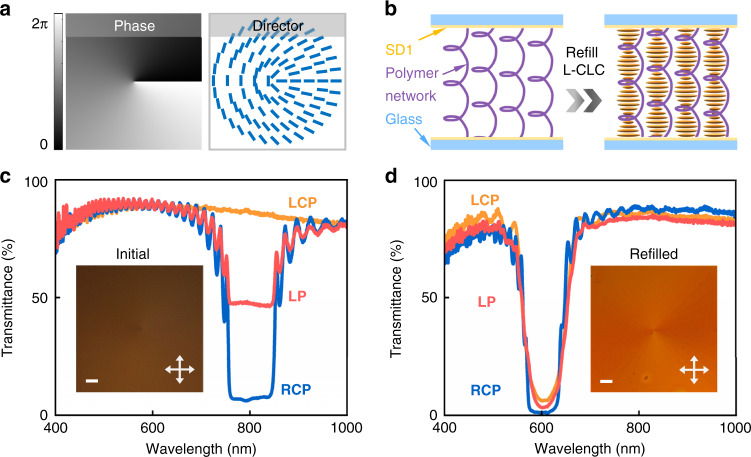
The designed pattern, refilling process, and transmittance spectra of opposite-chirality-coexisted superstructures for spin-dependent OVs. **a** Spiral phase pattern with *q* = +0.5 and corresponding surface LC director distribution. The color variation from black to white denotes the phase varying from 0 to 2π. The short blue stick indicates the local LC director on the initial side of CLC helixes. **b** Schematic illustration of the washed-out state (left) and the refilled state (right) of the fabrication process of opposite-chirality-coexisted superstructures. **c**, **d** The transmittance spectra of the initial state and the refilled state under different incident polarization of LCP (orange), LP (pink), and RCP (blue), respectively. Inserts are corresponding reflective micrographs observed under a polarized optical microscope. Crossed white arrows represent the orthogonal polarizer and analyzer. All scale bars are 100 μm

If somehow chiral superstructures with contrary handedness could be integrated into a single layer to form a uniformly-distributed and sub-wavelength local chirality heterogeneity, namely, the opposite-chirality-coexisted superstructures, light beams with orthogonal circular polarization and conjugated PB phase should be simultaneously reflected and superposed ($\left| {{{\mathbf{R}}}} \right\rangle {{{\mathrm{e}}}}^{ + i2\alpha } + \left| {{{\mathbf{L}}}} \right\rangle {{{\mathrm{e}}}}^{ - i2\alpha }$), as vividly shown in Fig. 1c. Take the typical spiral PB phase as an instance. LC directors (*α*) on the initial side of CLC helixes should rotate in accordance with the azimuthal angle (*θ*) following *α* = *qθ* + *α*_0_^38^, where *q* is half of the topological charge (*m*) of generated OVs and *α*_0_ is the initial angle (e.g., *q* = +0.5 and *α*_0_ = 0 in Fig. 2a). Through imprinting such azimuthally variant helixes into the opposite-chirality-coexisted superstructures, the RCP/LCP will be reflected and respectively transformed to the OV with a conjugated spiral phase of e^+*i*2*qθ*^/e^-*i*2*qθ*^. Particularly, for LP incident, it could be wholly reflected, contributing to the superposition of OVs with contrary topological charge and orthogonal circular polarization ($\left| {{{\mathbf{R}}}} \right\rangle {{{\mathrm{e}}}}^{ + i2q\theta } + \left| {{{\mathbf{L}}}} \right\rangle {{{\mathrm{e}}}}^{ - i2q\theta }$), namely, the cylindrical vector beam^39^. This can hardly be achieved by PB phase modulation of traditional CLCs, and more complicated functionalities can be rationally expected.

### Construction of opposite-chirality-coexisted superstructures

Here, the wash-out-refill process^36,40,41^ is adopted to construct the opposite-chirality-coexisted superstructures (Fig. S1). Firstly, two glass substrates spin-coated with photo-alignment agent SD1^9^ (see its chemical structure in Fig. S2) were assembled to form a cell. Then, the multi-step partly overlapping exposure^41^ was carried out to get the desired surface LC director distribution. Secondly, the CLC mixture composed of nematic LC, right-handed chiral dopant, and reactive monomers (see chemical structures in Figs. S2 and S3) was filled into the photo-patterned empty cell. Due to the surface anchoring effect of the photo-alignment agent to adjacent LC molecules and the chiral self-assemble capability of CLCs, a right-handed CLC superstructure was constructed with the desired azimuthally variant helixes. Thirdly, by irradiation with UV light (20 mW cm-2), the functional groups of orderly arranged monomers were crosslinked to form the polymeric backbone^34^. After each side of the 8-μm-thick cell was irradiated for 10 min, respectively, the uniform polymer network was formed and preserved the chirality of the original right-handed CLC. The corresponding optical structure was also frozen, retaining the optical properties and functionalities. Fourthly, this partly polymerized sample was immersed in acetone for 24 h to wash out the nonreactive LC molecules and unreacted monomers. After drying at 100 °C for 3 h, the obtained right-handed polymer network was refilled by the opposite-handed CLC (Fig. 2b), which was composed of nematic LC E7 and left-handed chiral dopant S5011 at a weight ratio of 97.7:2.3. Finally, the opposite-chirality-coexisted superstructure was achieved, preserving the initial surface director distribution.

Transmittance spectra and reflective polarized optical micrographs are recorded after each step of the fabrication procedure. When the right-handed CLC mixture is filled into the empty cell (i.e., the initial state), an appropriate Grandjean planar texture is observed as inserted in Fig. 2c. Its transmittance spectra under different incident polarization show that only RCP is reflected within the photonic band gap, while LCP is transmitted, resulting in about half transmittance for LP. After UV polymerization (i.e., the polymerized state), no obvious change is observed in its texture, and the RCP-selectivity remains (Fig. S4a). A little blue shift of the reflection band is attributed to the crosslinking-induced volume shrinking^33,42^. After washing out the unreacted molecules (i.e., the washed-out state), the reflective micrograph turns dark because the spin-selective reflectance of the polymer network vanishes in the visible region (Fig. S4b). Notably, by further refilling the opposite left-handed CLC into such a right-handed polymer network (i.e., the refilled state), not only the RCP but also the LCP is reflected within the identical wavelength range, exhibiting a polarization-independent photonic band gap with a high reflectance up to 95% (Fig. 2d). Few experimental deviations from the theory in some transmittance spectra are mainly induced by the fabrication error and the instrumental error related to the achromatic quarter-wave plate. Compared to that of the initial state (Fig. 2c), the central wavelength in Fig. 2d blue shifts from 803 nm to 608 nm together with a narrowed bandgap from 86 to 63 nm correspondingly. This mainly results from the incomplete reoccupation and the pitch shrinkage during the washing and the drying process^43,44^. It is worthy to note that the final transmittance spectra are affected by the fabrication process, including the washing time in acetone, the temperature and time of heating, and the refilled CLC (see more examples under different experimental conditions in Fig. S5). Especially, in order to obtain the polarization-independent high reflectance, the component ratio (E7/S5011) of the refilled CLC should be carefully adjusted to make its reflection band coincide with that of the polymer network. As inserted in Fig. 2d, the Grandjean planar texture with a central singular point is vividly observed, whose bright color is consistent with the transmittance spectra. This high reflectance of opposite polarization helicity breaks the inherent impression that CLC can only modulate single circular polarization, indicating the capability of PB phase reversal by altering the input SAM.

The CLC composite after refilling can be seen as a chiral polymer network surrounded by the CLC with the opposite handedness. Here, to spare enough space for the refilled CLC to preserve its chirality, the ratio of reactive monomers is carefully adjusted to a low value of 20.4%, where macromolecules (RM257/RM82) can form a network structure and small molecules (RM021/RM006/RM010) can blend into the CLC molecules and hold the chiral structure. Therefore, after washing and refilling, numerous distinct sub-wavelength domains coexist with either strongly network-dominated LCs or bulk CLCs^42^. For the domains far from the polymer network, the enclosed LC behaves much the same as in the bulk and takes on its preferred left-handed form, contributing to the LCP selective reflection. While, for the domains surrounding the right-handed polymer network, the LC takes the chirality of the network due to the anchoring effect, contributing to the RCP selective reflection. This association leads to the polarization-independent high reflectance^42^. It is important to note that the obtained local chirality heterogeneity is at the sub-wavelength scale and uniform across the whole single cell^45^. Thanks to this unique configuration, an average effect similar to the interleaving concept of optical metasurfaces can be introduced, resulting in the fantastic simultaneous PB phase modulation for both circular polarizations.

### Spin-dependent OVs

**Fig. 3:**
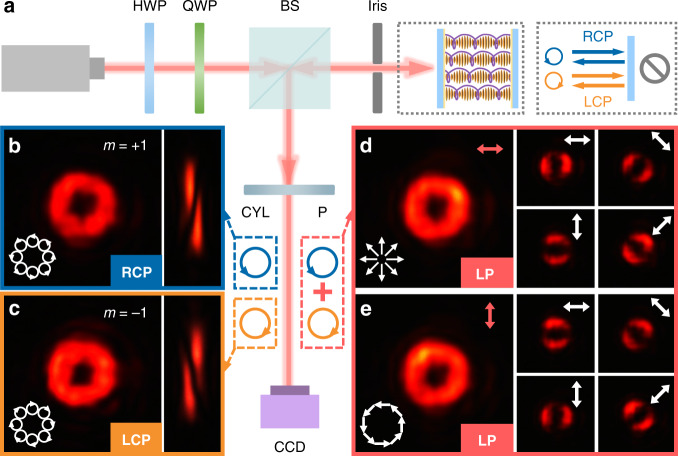
Generation and detection of OVs from the opposite-chirality-coexisted CLC sample. **a** Optical set-up. HWP half-wave plate, QWP quarter-wave plate, BS beam splitter, CYL cylindrical lens, P polarizer. Intensity profiles and polarization distributions of the reflected light at 610 nm under different incident polarization: **b** RCP (blue circle) and **c** LCP (orange circle) with corresponding OAM detection result, **d** horizontal LP and **e** vertical LP (pink double-ended arrows) with diffraction patterns after transmission through a rotating analyzer (white double-ended arrows)

Figure 3a illustrates the experimental optical setup to characterize the fabricated opposite-chirality-coexisted CLC sample. The half-wave plate and quarter-wave plate are used to control the incident polarization at 610 nm. After passing through the beam splitter, the incident light projects towards the sample and then gets reflected. Another polarizer is utilized to analyze the output polarization distribution. To verify the topological charges of generated OVs, the astigmatic transformation method^46^ is adopted by inserting a cylindrical lens (*f* = 100 mm) in front of the CCD. For the RCP incident, a donut-like intensity profile is obtained as shown in Fig. 3b. The corresponding converted pattern in the focal plane of the cylindrical lens is also presented. The number of dark stripes and their tilt direction indicate the topological charge *m* = +1. While for LCP incident, an OV with *m* = −1 is generated with high efficiency of over 92% (Fig. 3c). This verifies our expectation that the orthogonal spin-selective PB phase can be simultaneously modulated via such opposite-chirality-coexisted superstructures. Although with a little larger haze, the performances of the proposed device approach that of conventional single-chirality CLC device for circularly polarized light. Furthermore, for the horizontal LP, a radially polarized vector beam is obtained (Fig. 3d), which can be seen as the superposition of OVs with orthogonal circular polarization and contrary topological charge of *m* = ±1^39^. The intensity minima remain perpendicular to the transmission direction of the rotating analyzer. Similarly, an azimuthal polarization is achieved with vertical LP incident, whose intensity minima are parallel to the analyzer orientation (Fig. 3e). By changing the incident polarization, any other superposed state on the same higher-order Poincaré sphere^47^ can be generated as well. Such opposite-chirality-coexisted superstructures can easily realize the superposition of the conjugated PB phase, providing a compact platform for arbitrary reflective phase modulation.

### Advanced reflective PB phase modulation

**Fig. 4:**
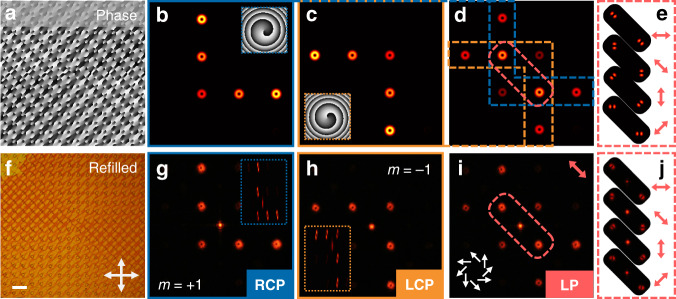
Simulations and experimental demonstration of the OV hologram by opposite-chirality-coexisted superstructures. **a** Designed phase pattern and **f** corresponding reflective micrograph of the refilled state. The scale bar is 100 μm. **b**–**e** Simulated and **g**–**j** experimental diffraction patterns of the sample under different incident polarization: **b**, **g** RCP, **c**, **h** LCP, and **d**, **e**, **i**, **j** 135° LP, respectively. The phase profile of an OV order and the converted diffraction patterns after the cylindrical lens are inserted in **b**, **c** and **g**, **h**, respectively. The polarization distribution of the vector beams in the marked orders is inserted in **i**. **e**, **j** Marked diffraction patterns with different orientations of the analyzer

To verify the powerful capability of the opposite-chirality-coexisted superstructures, OV holograms capable of reconstructing OV arrays are further demonstrated. By precisely designing the position and the topological charge of each OV and algebraically adding their complex amplitudes, we can calculate the desired phase hologram, and then get the desired LC director distribution. For the targeted multichannel OV arrays, the total complex field (*E*_tot_) at the hologram plane should be formulated as: $E_{{{{\mathrm{tot}}}}}(m,k_x,k_y) = \mathop {\sum}\limits_{j = 1}^N {A_j{{{\mathrm{e}}}}^{im_j\theta }} e^{ - i\left( {k_{xj}x + k_{yj}y} \right)}$^48^. *N* represents the total number of OVs. *k*_*xj*_ and *k*_*yj*_ are the wave vectors determining the diffraction angle of each OV in the *x*–*y* plane. *A*_*j*_ is the amplitude of OV with the topological charge *m*_*j*_. Accordingly, the phase pattern of OV hologram is $\psi = {{{\mathrm{arg}}}}\left( {E_{{{{\mathrm{tot}}}}}(m,k_x,k_y)} \right)$, and the director distribution encoded into CLC chiral superstructure follows $\alpha = \psi /2$. An example of the calculated phase pattern is presented in Fig. 4a, whose simulated diffraction results are exhibited in Fig. 4b–e. For RCP incident, an English alphabet “L” is constructed by five OVs with *m* = +1 and anticlockwise phase profile (Fig. 4b). While for LCP, an Arabic numeral “7” composed of OVs with *m* = −1 is shown in Fig. 4c. Figure 4d depicts the LP incident case where the reconstructed images in Fig. 4b, c are linearly superimposed. Accordingly, two overlapped orders turn to the vector beams with their polarization distributions verified in Fig. 4e.

Through the same fabrication process, this hologram pattern can be imprinted into the opposite-chirality-coexisted superstructures. The reflective micrographs of the initial, polymerized, washed-out, and refilled state are presented in Figs. S6 and 4f, respectively. The refilled state presents a planar texture with regular patterns consistent with Fig. 4a and the same color as Fig. 2d. The experimental diffraction images are presented in Fig. 4g, j, which are without obvious scattering and match well with the simulated results (Fig. 4b–e). For RCP and LCP incidents, the topological charge distribution of the OV array is detected by the cylindrical lens as inserted in Fig. 4g, h, respectively. Thanks to the merit of PB phase reversal, vector beams can be created in the overlapped positions via inputting LP. For instance, the spiral vector beam is obtained by rotating the incident LP to 135°, and the intensity minima form an angle of 45° with respect to the transmission axis of the analyzer (Fig. 4j). The cases of radial and azimuthal polarization can be found in Fig. S7. The faithful construction of these delicate chiral superstructures and the successful modulation of the complex PB phase verify the enormous possibilities of the proposed opposite-chirality-coexisted system.

## Discussion

In conclusion, the intrinsic limitation of traditional CLCs in manipulating a single spin-selective PB phase has been broken by the photo-patterned opposite-chirality-coexisted superstructures. Orthogonal circular polarizations are simultaneously endowed with conjugated PB phases in a single cell. As a typical demonstration, reflective OVs and vector beams are generated in a high-efficiency and polarization-dependent way. A more delicate pattern of OV hologram can also be faithfully imprinted into such chirality-coexisted superstructures, and the conjugated OV arrays are reconstructed in the form of “L” or “7,” verifying the fantastic performances in wave-front manipulation. Compared to traditional CLC stacks or mirror-backed CLC devices^31^, the proposed method exhibits some important merits of ultra-compact device configuration, exemption from careful alignment, and higher efficiency without multiple interfaces. Besides, the single-layer configuration has great potential to significantly decrease the wavelength-dependent propagation phase difference between RCP and LCP of polychromatic light within stacking layers. Such an open-ended strategy of opposite-chirality-coexisted anisotropic media brings important insight into the understanding of PB phase and polarization optics and facilitates the architectures and functionalities of soft chiral superstructures toward versatile elegant photonic devices.

## Materials and methods

### Materials

The UV polymerizable CLC mixture is composed of nematic LC (E7, HCCH, China), right-handed chiral dopant (R5011, HCCH, China, see its chemical structure in Fig. S2), LC reactive monomers (HCCH, China), and photo-initializer Diphenyl ketone (Shanghai Lingfeng Chemical Reagent Co. Ltd, China) at a weight ratio of 76.9:1.7:20.4:1. LC reactive monomers are composed of RM021, RM257, RM82, RM006, and RM010 (see respective chemical structures in Fig. S3) at a weight ratio of 20:30:15:20:15. This mixture was stirred magnetically at 1000 rpm at 70 °C for 30 min to be sufficiently blended. Besides, a UV-polarization-sensitive sulfonic azo-dye SD1 (Dai-Nippon Ink and Chemicals, Japan, see its chemical structure in Fig. S2) was dissolved in dimethylformamide at a concentration of 0.3 wt% and used as the photo-alignment agent. SD1 molecules tend to reorient their absorption oscillators perpendicular to the polarization direction of the illuminating UV light and further guide LCs^9^.

### Photo-patterning process

Indium-tin-oxide glass substrates (1.5 × 2 cm^2^) were ultrasonically bathed, UV-Ozone cleaned, and then spin-coated with the SD1 layer. After curing at 100 °C for 10 min, two pieces of glass substrates were assembled to form an 8-μm-thick cell with the epoxy glue. Then, it was placed at the image plane of the digital-micro-mirror-device (DMD)-based dynamic photo-patterning system. Briefly speaking, the surface director distribution of each pattern was calculated and divided into 36 sub-regions endowed from 0° to 175° at an interval of 5°. A sum of five adjacent sub-regions was exposed simultaneously and the subsequent exposure shifted one sub-region with the polarizer rotating 5° synchronously. Finally, each sub-region was exposed five times with a total exposure dose of 5 J cm^−2^. Through such a 36-step 5-time partly-overlapping exposure process^46^, the designed director distributions can be carried out on the substrates. Then, the right-handed CLC mixture was filled into the photo-patterned empty cell at 70 °C by the capillarity action and gradually cooled to room temperature.

### Characterizations

All characterizations were performed at room temperature under an ambient environment. All micrographs were recorded under the reflective mode of an optical microscope (Ci POL, Nikon, Japan) with crossed polarizer and analyzer. The transmittance spectra were measured with a halogen light source (iDH2000H-HP, ideaoptics, China), the achromatic quarter-wave plate, the broadband polarizer, and a spectrometer (PG2000-Pro-EX, ideaoptics, China). The supercontinuum fiber laser (SuperK EVO, NKT Photonics, Denmark) was filtered at different monochromatic wavelengths by the multi-channel acousto-optic tunable filter (SuperK SELECT, NKT Photonics, Denmark). Reflected diffraction patterns were captured by a CCD camera (DCC1645C-HQ, Thorlabs, USA) or a digital camera (EOS M, Canon, Japan).

## Supplementary information

Supplementary Information for Pancharatnam-Berry phase reversal via opposite-chirality-coexisted superstructures

## Data Availability

The data that support the findings of this study are available from the corresponding author upon reasonable request.
