# Supplementary material for: Pancharatnam–Berry phase reversal via opposite-chirality-coexisted superstructures
Source: Light Sci Appl. 2022 May 12;11:135. doi: 10.1038/s41377-022-00835-3 (PMC9098607; doi:10.1038/s41377-022-00835-3)
Supplement: Supplementary file 1 — Supplementary Information for Pancharatnam-Berry phase reversal via opposite-chirality-coexisted superstructures [file 41377_2022_835_MOESM1_ESM.pdf]

Supplementary Information for

Pancharatnam-Berry phase reversal via opposite-chirality-coexisted superstructures

Lin Zhu,[#] Chun-Ting Xu,[#] Peng Chen,* Yi-Heng Zhang, Si-Jia Liu, Quan-Ming Chen, Shi-Jun Ge, Wei Hu, and Yan-Qing Lu*

National Laboratory of Solid State Microstructures, Key Laboratory of Intelligent Optical Sensing and Manipulation, College of Engineering and Applied Sciences, and Collaborative Innovation Center of Advanced Microstructures, Nanjing University, Nanjing 210093, China.

[#]**These authors contributed equally:** Lin Zhu, Chun-Ting Xu.

***Correspondence:** Peng Chen (chenpeng@nju.edu.cn), and Yan-Qing Lu (yqlu@nju.edu.cn)

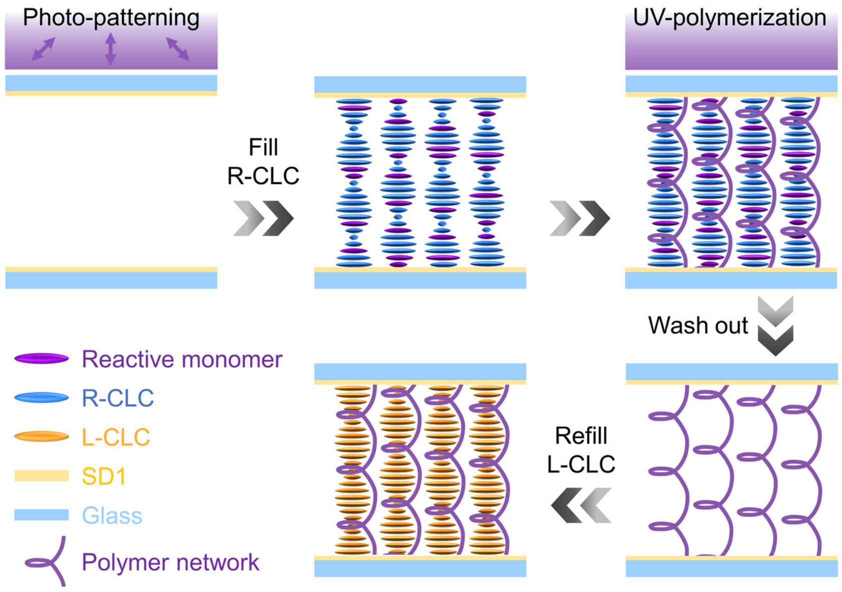

Fig. S1 The fabrication process of the photo-patterned opposite-chirality-coexisted superstructures. R-CLC, right-handed CLC; L-CLC, left-handed CLC.

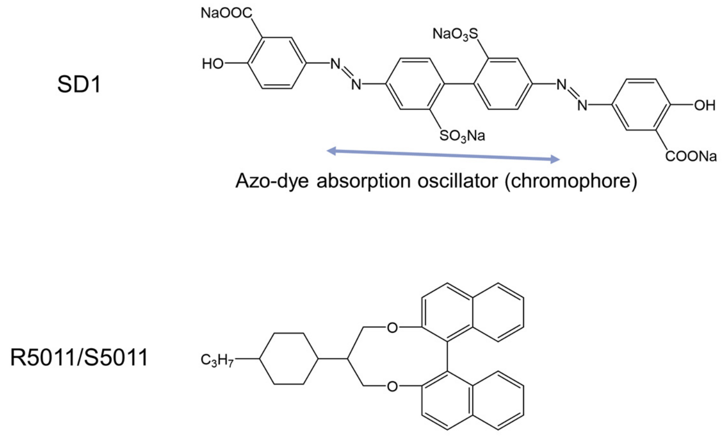

Fig. S2 Molecule structures of the photoalignment agent SD1 and chiral dopant R5011/S5011.

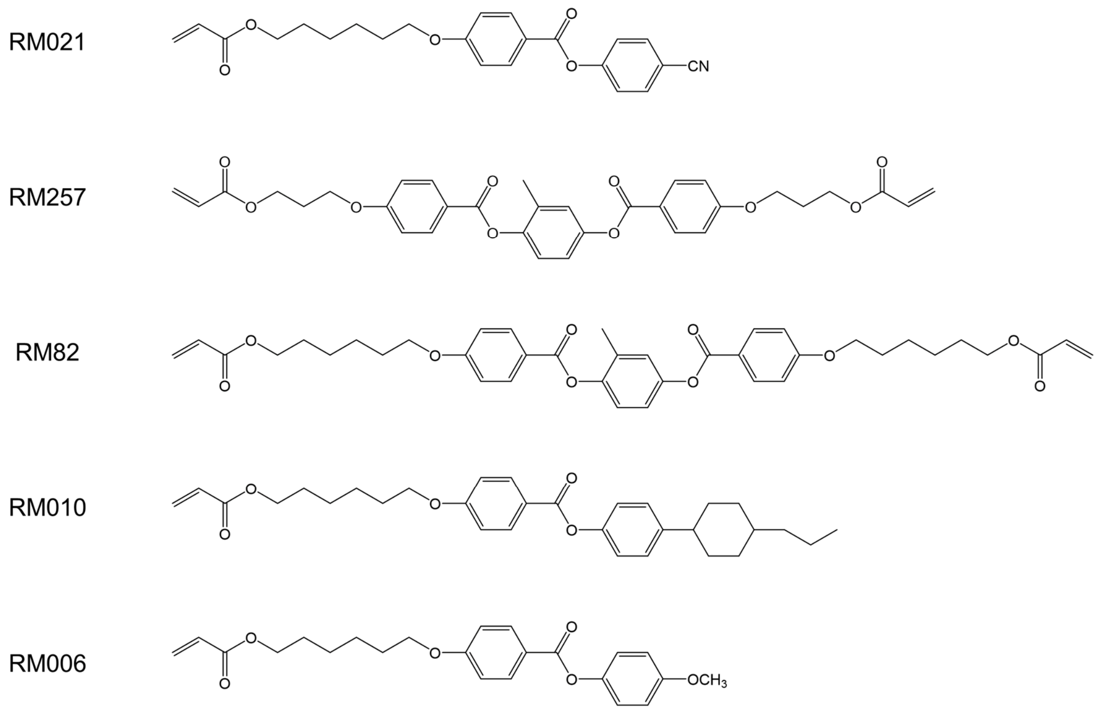

Fig. S3 Molecule structures of the utilized reactive monomers.

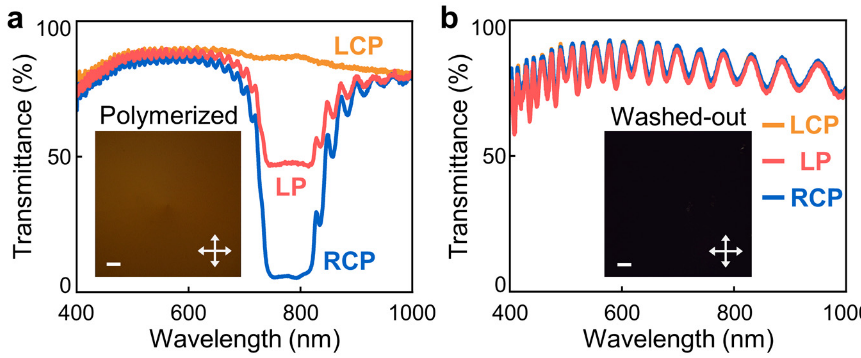

Fig. S4 The transmittance spectra and reflective micrographs. a The polymerized state and **b** the washed-out state of OV sample under different incident polarization of LCP (orange), LP (pink), and RCP (blue), respectively. Inserts are corresponding reflective micrographs observed under a polarized optical microscope. All scale bars are 100 μm.

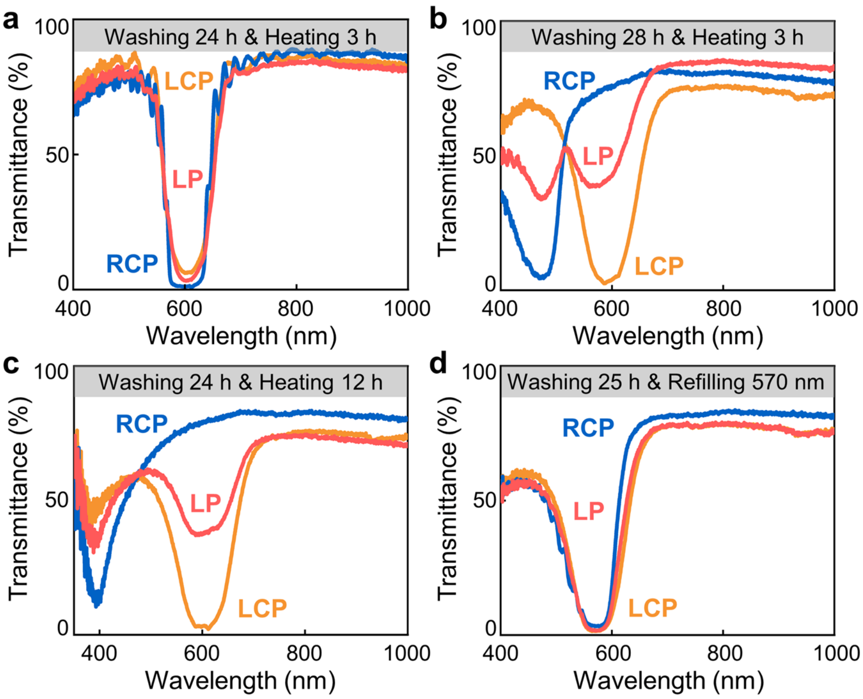

Fig. S5 The transmittance spectra under different conditions of washing, heating, and refilling.

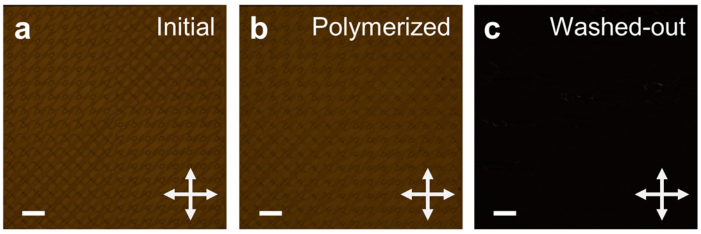

Fig. S6 Reflective micrographs. a The initial state, **b** the polymerized state, and **c** the washed-out state of the hologram sample. All scale bars are 100 μm.

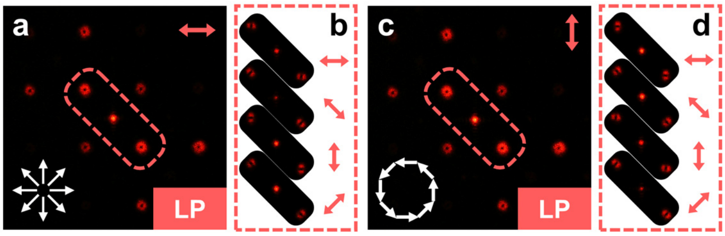

Fig. S7 Diffraction patterns of the hologram sample under different incident polarization. a, b The horizontal LP; **c, d** the vertical LP. White arrows indicate the polarization distribution of vector beams in marked orders of **a** radial and **c** azimuthal polarization, with polarization detection results shown in **b** and **d**, respectively.
